# Hematopoietic Cells Influence Vascular Development in the Retina

**DOI:** 10.3390/cells11203207

**Published:** 2022-10-13

**Authors:** Bright Asare-Bediako, Yvonne Adu-Agyeiwaah, Antonio Abad, Sergio Li Calzi, Jason L. Floyd, Ram Prasad, Mariana DuPont, Richmond Asare-Bediako, Xose R. Bustelo, Maria B. Grant

**Affiliations:** 1 Vision Science Graduate Program, School of Optometry, University of Alabama at Birmingham, Birmingham, AL 35233, USA; 2 Department of Ophthalmology and Visual Sciences, School of Medicine, University of Alabama, Birmingham, AL 35294, USA; 3 Centro de Investigación del Cáncer de Salamanca, CSIC and University of Salamanca, 37007 Salamanca, Spain; 4 Instituto de Biología Molecular y Celular del Cáncer, CSIC and University of Salamanca, 37007 Salamanca, Spain; 5 Centro de Investigación Biomédica en Red de Cáncer (CIBER), CSIC and University of Salamanca, 37007 Salamanca, Spain; 6 Thomas H. Gosnell School of Life Sciences, Rochester Institute of Technology, Rochester, NY 14623, USA

**Keywords:** hematopoietic cells, developing retina, vasculogenesis, microglia, Vav1 knockout

## Abstract

Hematopoietic cells play a crucial role in the adult retina in health and disease. Monocytes, macrophages, microglia and myeloid angiogenic cells (MACs) have all been implicated in retinal pathology. However, the role that hematopoietic cells play in retinal development is understudied. The temporal changes in recruitment of hematopoietic cells into the developing retina and the phenotype of the recruited cells are not well understood. In this study, we used the hematopoietic cell-specific protein Vav1 to track and investigate hematopoietic cells in the developing retina. By flow cytometry and immunohistochemistry, we show that hematopoietic cells are present in the retina as early as P0, and include microglia, monocytes and MACs. Even before the formation of retinal blood vessels, hematopoietic cells localize to the inner retina where they eventually form networks that intimately associate with the developing vasculature. Loss of Vav1 lead to a reduction in the density of medium-sized vessels and an increased inflammatory response in retinal astrocytes. When pups were subjected to oxygen-induced retinopathy, hematopoietic cells maintained a close association with the vasculature and occasionally formed ‘frameworks’ for the generation of new vessels. Our study provides further evidence for the underappreciated role of hematopoietic cells in retinal vasculogenesis and the formation of a healthy retina.

## 1. Introduction

Hematopoietic cells are bone-marrow derived cells that become blood-borne myeloid and lymphoid cells and migrate to various tissues, participating both in immune responses and the maintenance of specific tissues [1,2,3]. Produced in the bone marrow in the adult homeostatic state [4], hematopoietic cells consist of undifferentiated, multipotent stem and progenitor cells responsible for their self-renewal and downstream mature immune cells [5]. Hematopoietic cells are involved in the maintenance of immune homeostasis in health [6] but are also activated in disease or stress to fight infection and aid in tissue repair. For example, in the steady state, monocytes patrol the vascular wall to assist in removal of infectious agents and other pathogens [7,8]. During injury or disease however, monocytes are recruited into the tissue parenchyma where they differentiate into macrophages with proinflammatory and anti-inflammatory activities [9,10,11]. In the adult retina, hematopoietic cells are immediately recruited following acute injury and home to sites of injury to facilitate tissue repair [2]. In the diabetic retina, hematopoietic cells accumulate in the lumen of the vasculature in response to increased vascular inflammation leading to capillary non-perfusion, endothelial cell loss and the eventual formation of acellular capillaries [12,13,14] and also extravasate from vessels to promote additional retinal pathology.

In murine development, hematopoietic cells first appear in the embryonic yolk sac between embryonic days 7 (E7) and 8 (E8) [15,16]. They are later detected in the aorta–gonads–mesonephros (E10.5), fetal liver (E11.5) and then the bone marrow (E14–16.5), which becomes the dominant hematopoietic site in the adult state. Concurrently, retina development begins around E10 and matures postnatally [17,18]. Multiple studies have shown that some resident immune cells in the retina, typically microglia, are seeded in the eye from hematopoietic cells in the embryonic yolk sac, which migrate and proliferate in the retina [19,20]. However, a detailed characterization of the different phenotypes of hematopoietic cells in the developing retina is lacking, and the role that these cells play in retina development is understudied. The goal of this study was to investigate the timeline of the migration of different hematopoietic cells into the developing mouse retina postnatally and their contribution to the formation of a healthy adult retina.

## 2. Materials and Methods

### 2.1. Animals

All animal experiments adhered to the Association for Research in Vision and Ophthalmology Statement on the Use of Animals in Ophthalmic and Visual Research and were approved by the Institutional Animal Care and Use Committee of University of Alabama at Birmingham (APN-21223), the Bioethics Committee of the University of Salamanca (animal license #568) and the animal experimentation authorities of the autonomous Government of Castilla y León (Spain). They were treated humanely in accordance with standards described in the Guide for the Care and Use of Laboratory Animals, considering relevant national and European guidelines.

To generate Vav1-GFP mice, ROSA^mTmG^ mice (Gt(ROSA)26Sortm4(ACTB--tdTomato,-EGFP)Luo/J; The Jackson Laboratory, Bar Harbor, ME, USA) were crossed with Vav1-icre (B6.Cg-Commd10Tg(Vav1-icre)A2Kio/J; The Jackson Laboratory) mice. This allowed the expression of GFP under the Vav1 promoter, giving rise to GFP+ (Vav1) hematopoietic cells in subsequent progeny. Vav1-GFP newborns were euthanized at P0, P7, P14 and P21 and their retinas used for experiments and analysis. Vav1^−/−^ (Vav1 KO) mice were generated as described previously [21,22].

### 2.2. Flow Cytometry

Mice were perfused transcardially under isoflurane anesthesia with phosphate-buffered saline (PBS) to flush vessels before euthanasia. After euthanasia, eyes were enucleated into ice-cold phosphate-buffered saline (PBS). The corneas, lenses and hyaloid vasculatures were removed and the retinas isolated immediately. To dissociate retinas into single cells, each retina was incubated in 1 ml of papain dissociation solution (Roche Diagnostics GmbH, REF#10108014001, Mannheim, Germany) prepared according to manufacturer’s protocol for 30 min in a 37 °C water bath. Single cell suspensions were washed with FACs buffer on ice and then incubated with primary antibody cocktails for 45 min at 4 °C in the dark. Antibodies used included CD45 Apc-eFluor780 (Invitrogen, Cat#47-0451-82, Waltham, MA, USA), Ly6C Percp/Cy5.5 (Biolegend, Cat#128012, San Diego, CA, USA), Ly6G (BV605 Biolegend, Cat#127639), CD11b PE-CF594 (BD Biosciences, Cat#562287, Franklin Lakes, NJ, USA), CD31 PE (Invitrogen, Cat#2114546) and Fixable viability Dye eFluor506 (Invitrogen, Cat# 65-0866-14). The cells were then washed with FACs buffer, resuspended and analyzed using BD FACSCelesta flow cytometer (BD Biosciences) and FlowJo™ v10.8 Software (BD Life Sciences, Franklin Lakes, NJ, USA). Gating of the different cell populations was performed as previously published [23].

### 2.3. Immunofluorescence of Flat-Mounted Retinas and Retinal Cross-Sections

Immunofluorescence of retinal cross-sections was performed as previously described [24]. Briefly, enucleated eyes were fixed in 4% paraformaldehyde solution for 15–30 min on ice. After removing the corneas and lenses, the posterior cups were incubated in 15% sucrose solution in phosphate-buffered saline (PBS) overnight at 4 °C, then transferred to 30% sucrose in PBS for 3–4 h. The samples were then embedded in optimal cutting temperature (O.C.T.) medium and immediately frozen on dry ice and stored at −80 °C until further processing. The sections were thawed at 37 °C for 15–30 min., washed in PBS and then permeabilized with 0.25% Triton-X in PBS for 5 min at room temperature. Sections were blocked with 10% normal horse serum in 1% bovine serum albumin (BSA) for 1 h and incubated with primary antibody diluted in blocking solution (1:100 dilution) overnight at 4 °C. Samples were then washed and incubated with the appropriate fluorescent-labeled secondary antibodies for 1 h at room temperature, followed by washing with PBS. Sections were incubated with 40,6-diamidino-2-phenylindole, dihydrochloride (DAPI) solution (Invitrogen, Cat#D3571) for 5 min at room temperature. For retinal flat mounts, enucleated eyes were fixed in 4% paraformaldehyde solution for 90 min on ice. After fixation, the corneas, lenses and hyaloid vasculatures were removed and the whole retinas isolated, washed in PBS and incubated in blocking buffer for 3 h at room temperature. The samples were then incubated in primary antibodies overnight at 4 °C, washed and incubated with secondary antibodies at room temperature for 4 h. The primary antibodies used were chicken anti-GFAP (Novus Biologicals, Cat# NBP1-05198, Centennial, CO, USA) and rabbit anti-collagen IV (Abcam, Cat#19808, Cambridge, UK). Finally, samples were washed and mounted with anti-fade mounting medium (Vector Laboratories, Cat# H-1000, Burlingame, CA, USA) for imaging. Images were obtained with a 40× objective lens for cross-sections and 20× for flat mounts and all experiments included negative controls in which the primary antibodies were replaced with blanks (blocking buffer). All analyzed images were acquired from the mid-periphery of the retina where we observed high density of vessels without interference from very large vessels at all time points.

### 2.4. Oxygen-Induced Retinopathy (OIR)

To study the impact of hematopoietic cells on abnormal retinal vascular development, we utilized the oxygen-induced retinopathy (OIR) model [25,26] as previously published [27]. Briefly, Vav1-GFP mice (P7) were placed with their nursing dams in a 75% oxygen atmosphere for 5 days. Mice were returned to normoxic conditions at P12 and euthanized at P15 and P17, the timepoint for peak neovascularization or angiogenesis [28,29]. Retinas of the OIR mice were used in flat mount preparations as detailed above.

### 2.5. Quantification of Retinal Vascular Density Using VESGEN

VESGEN is a JAVA-based vascular analysis software program available from NASA (https://software.nasa.gov/software/ARC-17621-1 (accessed on 5 September 2022)) and operates as an ImageJ plugin [30,31]. In addition to the assessment of overall vascular densities, VESGEN also allows grouping and quantification of different generations of blood vessels in retinal images. For this study, 20× images of retinal flat mounts were traced and binarized in Adobe Photoshop CC 2018 v19.1.2 (Adobe Systems Incorporated, San Jose, CA, USA). The resulting binary images were loaded into VESGEN2D v1.11 for analysis. For this murine study, large-sized (macrovascular) vessels were defined as generations 1–3, medium-sized vessels as generations 4–6 and small-sized (microvascular) vessels were defined as generations 7 and greater [32].

## 3. Results

### 3.1. Phenotype and Localization of Hematopoietic Cells in Healthy Developing Retina

**Figure 1:**
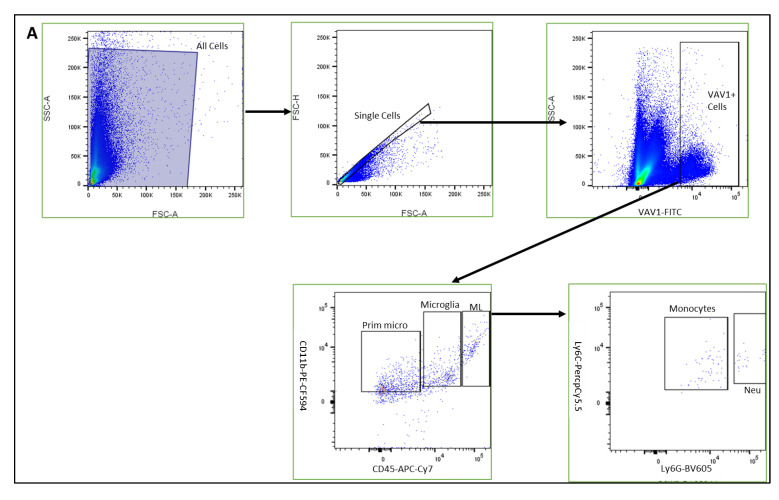

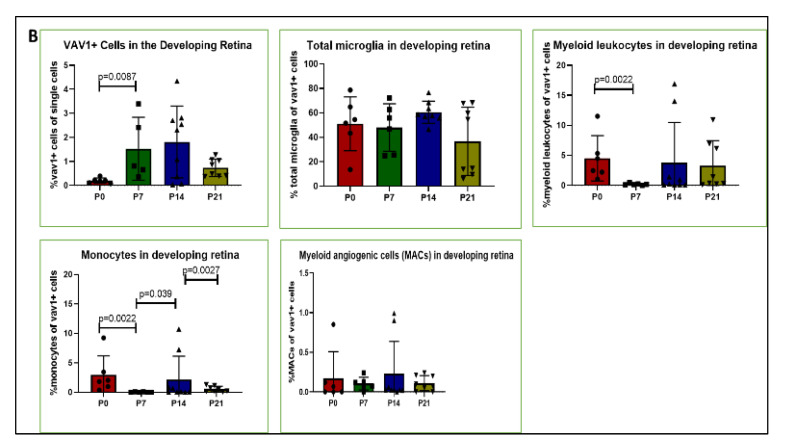
Hematopoietic cells in developing retina (**A**)**.** Representative plots showing flow cytometric analysis gates used to quantify cells within the retina. After gating for single cells, events were sub-gated into GFP^+^(Vav1) cells, GFP^+^/CD11b^+^/CD45^high^ myeloid leukocytes (ML), GFP^+^/CD11b^+^/CD45^low^ microglia and GFP^+^/CD11b^+^/CD45^−^ ‘primitive’ microglia. ML were sub-gated into Ly6C^+^Ly6G^−^ monocytes, and CD31^+^ myeloid angiogenic cells (MACs). Panel (**B**) shows quantification for each of these populations at P0, P7, P14 and P21 as indicated.

Using mice that express GFP under the control of the promoter for Vav1, a gene that encodes a hematopoietic cell-specific signaling protein [33,34,35,36], we investigated the phenotype of hematopoietic cells in the retina during post-natal development by flow cytometry and immunohistochemistry. As shown in Figure 1, GFP^+^ hematopoietic cells were detected in the retina less than 24 h after birth (P0). The proportion of these cells in the retina increased significantly after P7 but remained steady up to P21. We observed that the majority of GFP^+^ hematopoietic cells early in post-natal development were microglia (53.05%, 51.34%, 58.37%, for P0, P7, P14, respectively,). The proportion of hematopoietic cell-derived microglia was reduced at P21 (34.61%) but did not reach statistical significance (*p* = 0.161). In addition, we detected GFP^+^ myeloid angiogenic cells (MACs) and monocytes in the developing retina. While the levels of the angiogenesis-supporting MACs were steady from P0 to P21, we observed that the levels of monocytes fluctuated between early (P0 to P7), mid (P7 to P14) and late (P14 and P21) development.

**Figure 2:**
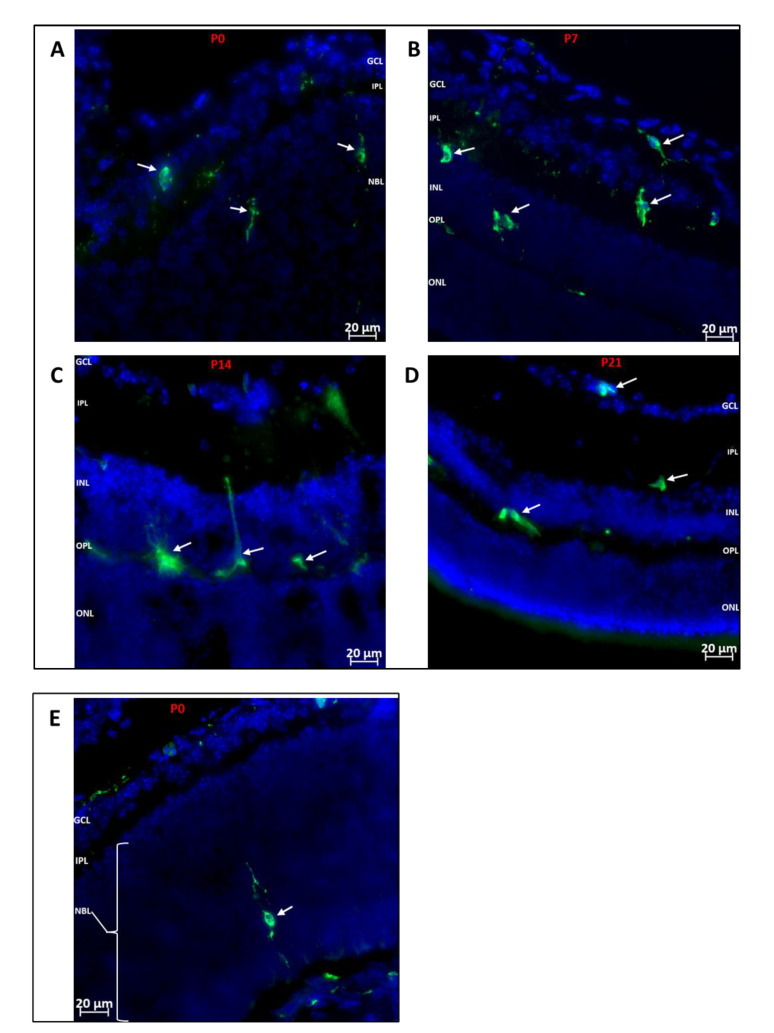
Localization of Hematopoietic Cells in Developing Retina (**A**–**D**)**:** Representative images showing localization of GFP^+^ (green) hematopoietic cells in the retina at P0, P7, P14 and P21. The cells predominantly migrate to the inner retina (inner limiting membrane to outer plexiform layer). (**E**): Representative image showing the occasional localization of a hematopoietic cell in the outer retina. A GFP^+^ cell is observed in the developing outer retina extending dendritic processes towards the RPE layer and inner retina. GFP^+^ cells are also observed arriving at the posterior retina via the choroid. Nuclei were stained with DAPI (blue). GCL: Ganglion cell layer; IPL: inner plexiform layer; NBL: neuroblast layer; INL: inner nuclear layer; OPL: outer plexiform layer; ONL: outer nuclear layer.

The post-natal development of the retina is a tightly controlled process, such that the different neuronal cell types are being specialized into mature neurons while vasculogenesis occurs concurrently. We investigated which retinal layers showed hematopoietic cell recruitment during development. As shown in Figure 2, we observed that GFP^+^ hematopoietic cells were recruited into the inner retina (from inner limiting membrane to outer plexiform layer) at all the time points investigated, while the outer retina was largely devoid of hematopoietic cells, except for dendritic processes of a few cells.

### 3.2. Hematopoietic Cells and Retinal Angiogenesis and Inflammation

**Figure 3:**
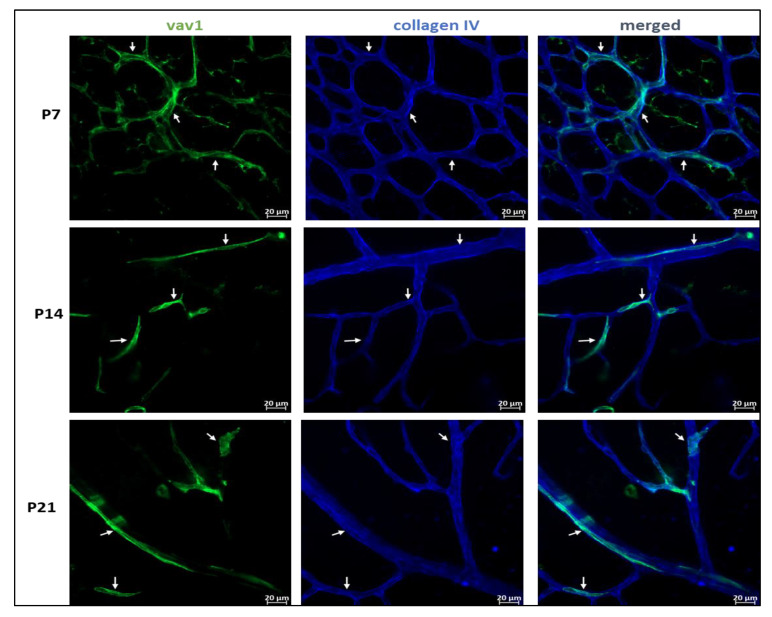
Hematopoietic cells and vascular development in the retina. Representative images showing the close association between GFP^+^ hematopoietic cells and the developing vasculature in the mouse retina. In early retinal vascular development (P7), hematopoietic cells in the retina form networks (P7, arrows) that parallel the developing vasculature, providing support to the vessels. Remnants of these networks persist throughout development (P14–P21, arrows).

**Figure 4:**
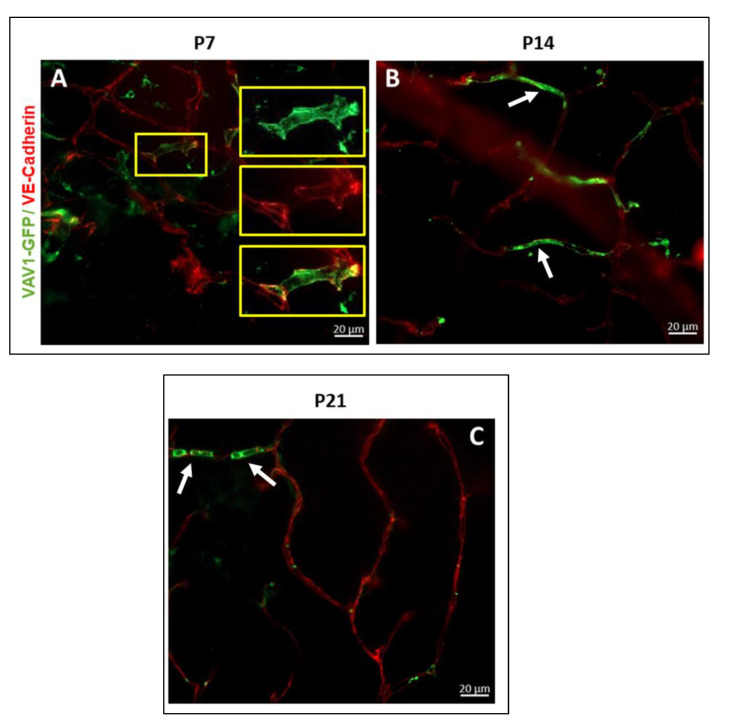
Hematopoietic cells in retinal vascular development. Hematopoietic cells support angiogenesis in the developing retina. GFP^+^ hematopoietic cells provide a framework for the completion of a branch vessel in the retina at P7 ((**A**), inset) and are occasionally incorporated into the developing vasculature at P14 ((**B**), white arrow) and P21 ((**C**), white arrow) for the formation of healthy/normal vessels.

One of the main events that occurs in the murine retina postnatally is the final formation of the vascular network. Using flat mounted retina and immunohistochemistry, we observed that GFP^+^ hematopoietic cells form networks that closely associated with the developing vasculature (Figure 3) and occasionally assimilated into vessels (Figure 4). At P7, we observed that GFP^+^ hematopoietic cells occasionally formed retinal vessels (Figure 4A, inset). This suggests that hematopoietic cells may play a role in retinal vasculogenesis during development.

**Figure 5:**
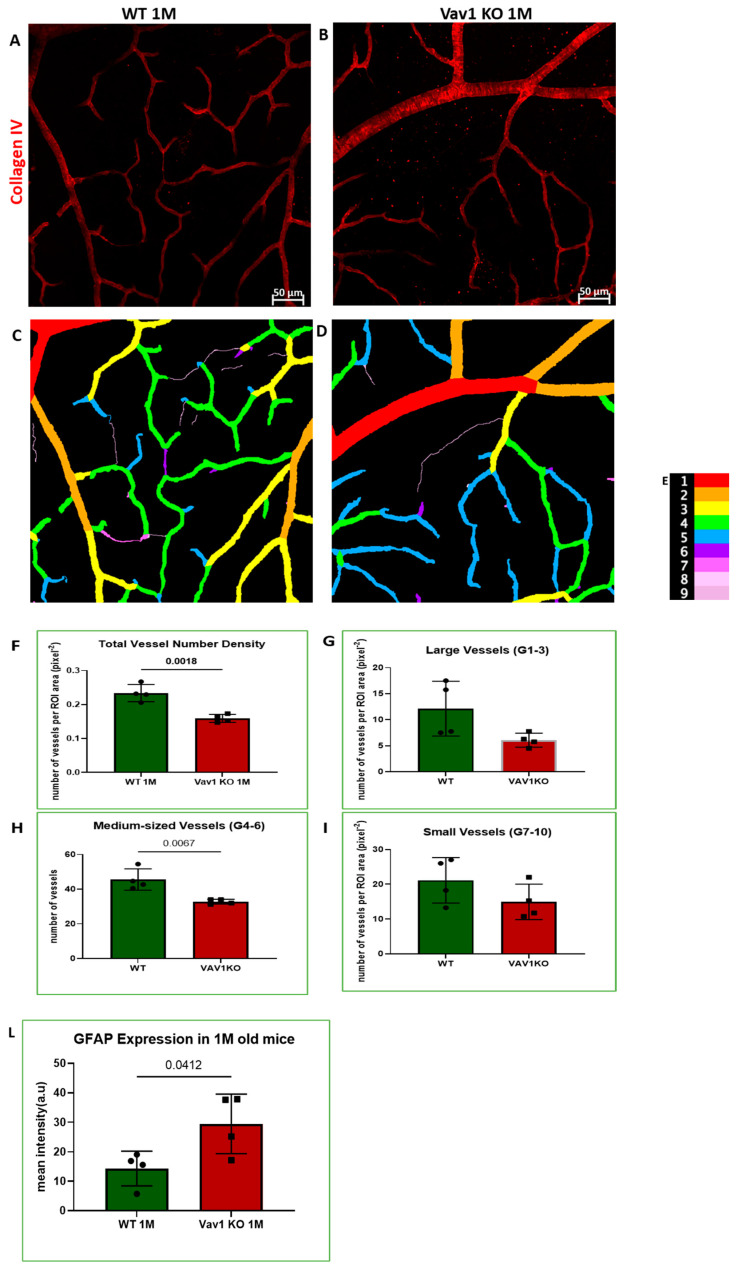

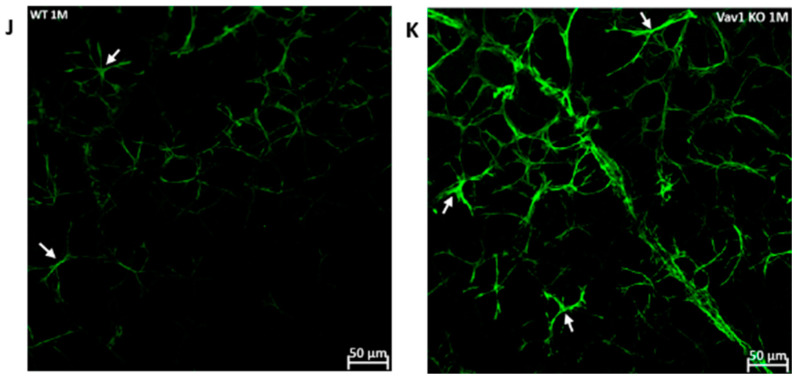
Loss of hematopoietic cells in the developing retina delays retinal vascular development and increases inflammation. Representative images (**A**,**B**) showing retinal flat mounts of WT and Vav1 KO retinas stained with collagen IV which labels the vasculature, and the corresponding outputs from VESGEN (**C**,**D**) showing the different vessel generations color coded (**E**) from 1–9. (**F**–**I**) Graphs showing VESGEN quantifications comparing total vessel number density, large vessel, medium-sized vessel and small vessel densities between the two groups. (**J**,**K**) Representative images showing GFAP expression (green) in the retinas of WT and Vav1 KO mice and the corresponding quantification in (**L**). White arrows indicate retinal astrocytes.

We next examined the retinas of Vav1 knockout (KO) mice to evaluate the impact of loss of functional hematopoietic cells on retinal vascular development and used VESGEN for analysis of the vasculature. As shown in Figure 5, we observed that Vav1 KO mice have a significantly reduced vessel number density at 1 month of age compared to wild-type (WT) mice (0.1592/pixel^2^ ± 0.011 vs. 0.2337/pixel^2^ ± 00025, *p* = 0.0018). We observed a significant difference in vessel density in the medium-sized vessels but not the large vessels or microvessels. We also examined the expression of glial fibrillary acidic protein (GFAP) in the developing retina. GFAP is normally expressed by retinal astrocytes but is increased with inflammation. We observed that GFAP expression was significantly elevated in Vav1 KO mice compared to WT (29.48a.u ± 10.09 vs. 14.34 ± 5.91, *p* = 0.0412), supporting an increased inflammatory response in retinal astrocytes of Vav1 KO mice. Finally, we examined 7-month-old Vav1 KO mice and found that they had reduced vascular density (Figure S2D) but not significant difference in GFAP expression (Figure S2E) compared to controls, suggesting that while the KO retinas recover from the increase in inflammation observed early after birth, the loss of vascular density persists.

### 3.3. Hematopoietic Cells in Abnormal Retinal Vascular Development

**Figure 6:**
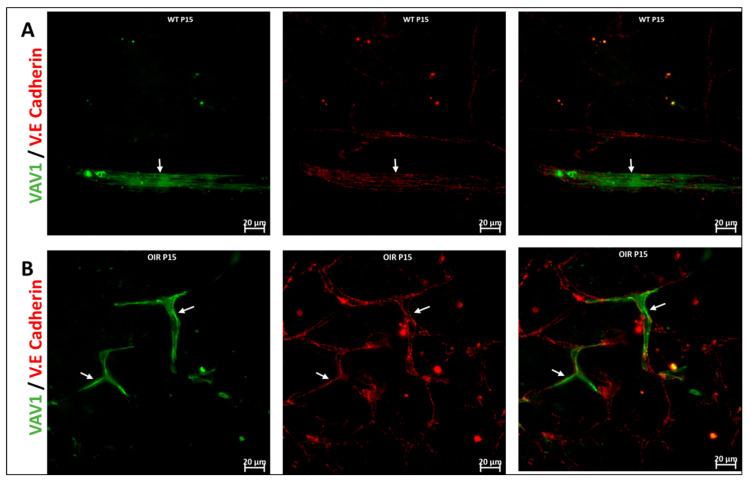

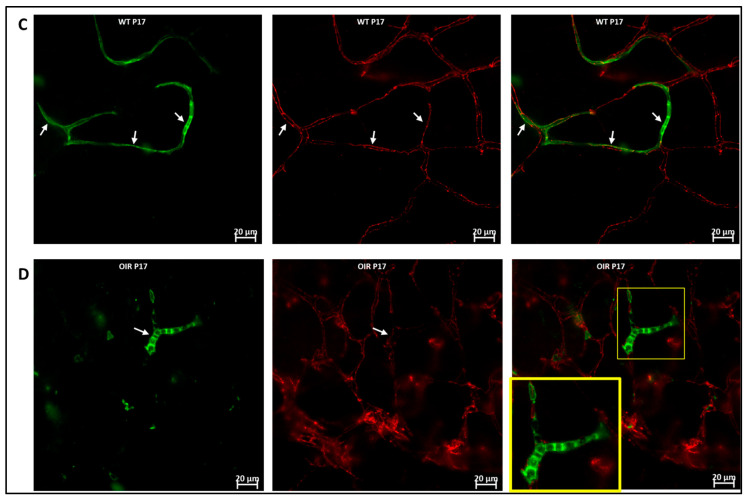
Hematopoietic cells in abnormal vascular development and repair. Representative images (**A**,**B**) showing retinal flat mounts of Vav1-GFP without OIR (**WT**) and with OIR (OIR) at 3 days (P15; (**A**,**B**)) and 5 days (P17; (**C**,**D**)) post OIR. At P15, GFP^+^ hematopoietic cells are observed forming close association with the vasculature in both models (white arrows). At P17, the peak of neovascularization, recruited GFP^+^ hematopoietic cells are observed to align/aggregate ((**D**)**,** inset) to remodel or repair a damaged vessel in the developing retina.

We used the OIR model to investigate the role of hematopoietic cells in retinal vascular repair during abnormal retinal development. In the OIR, exposure to high oxygen followed by a return to normoxia creates a relative hypoxic environment in the retina that leads to abnormal vascular development. Similar to WT retinas, we observed that GFP^+^ hematopoietic cells were recruited into the retina and were closely associated with the vasculature (Figure 6). In the retinas of mice subjected to OIR, hematopoietic cells aggregated and elongated to form vascular branches (Figure 6D) and formed new vessels or repaired damaged vessels at P17.

## 4. Discussion

The major findings of this work include that hematopoietic cells are recruited early in development and participate in the formation of the healthy retina mainly as microglia and by facilitating retinal angiogenesis. GFP^+^ cells in the retina appear as early as less than 24 h after birth (P0) and reached steady levels from P7 to P21 with the majority of these cells becoming microglia. We show that hematopoietic cells consistently align with the developing vasculature, potentially providing paracrine support to aid vasculogenesis in the healthy developing retina and also for vascular repair in abnormal vascular development.

The involvement of hematopoietic cells in maintenance of retinal health is widely appreciated in the adult retina. Hematopoietic cells are mobilized from the bone marrow and recruited into injured or into the diseased retina to orchestrate tissue repair, inflammation and/or cell death [37,38,39]. However, the role that these cells play in the development of the retina and the temporal dynamics of their recruitment during post-natal development is understudied. We used transgenic mice that express GFP in the hematopoietic cell-specific protein Vav1 [35] to study the temporal changes in the recruitment of hematopoietic cells into the developing retina postnatally. GFP^+^ hematopoietic cells appeared in the retina as early as P0 and reached steady levels from P7 to P21 (Figure 1). Retinal microglia were the primary cell type derived from hematopoietic cells. Microglia arise from primitive hematopoietic cells in the embryonic yolk sac (which become the resident microglia) and previous studies have identified microglia in the retina prenatally [10,20]. We observed myeloid leukocytes (ML) in the developing retina, and they were significantly reduced after P7. Of myeloid leukocytes in the retina, the levels of MACs remained consistent up to P21 while the number of monocytes fluctuated. The initial relatively high levels of ML (predominantly monocytes) are likely in response to neonatal stress [40,41] after exposure of the newborn to a new oxygenated environment, which influences the mobilization of hematopoietic cells [42]. Given that there is no blood-retinal barrier at birth in mice, circulating myeloid leukocytes enter the retina unrestricted. However, monocytes and other myeloid leukocytes only remain in the retina for a few days and are replaced [23]. Monocytes patrol the retina to protect against infection [7] and facilitate the activation of tissue resident microglia and macrophages to phagocytose [7,19] dead or misplaced cells as the developing retina organizes into distinct layers. The fluctuating levels of these cells in the developing retina is indicative of the dynamics of recruitment and removal of myeloid cells protecting the retina by facilitating the elimination of dead cells or unwanted cells every few days.

Whereas vascular wall-derived endothelial colony forming cells (ECFCs) are the main cells capable of forming blood vessels de novo, MACs are hematopoietic cells that provide paracrine support to ECFCs during angiogenesis [43,44]. Given that the newborn mouse retina is devoid of blood vessels, the MACs are likely recruited to facilitate formation of blood vessels de novo and their organization into layers in the developing retina postnatally. We observed that GFP+ hematopoietic cells were distributed over the entire retina without preference for central or peripheral retina as shown in Figure S1. Other studies have shown bone marrow-derived cells enter the retina through the optic nerve head and ciliary body [45]. With no blood vessels and no blood-retinal barrier until later in development, the cells enter the retina mainly through the optic nerve head prior to P0 (Figure S2A), and also through the choroid (as Figure 2E) and migrate towards the inner retina and periphery as vasculogenesis begins and progresses. The recruited hematopoietic cells predominantly occupied the inner retina, from the inner limiting membrane to the outer plexiform layer (Figure 2). Santos et al. [20] observed similar localization of microglia in the developing retina, which is what we observed in our study. Interestingly, normal vascular development in the mouse retina is confined to the inner retina [46,47]. We observed that GFP+ hematopoietic cells organized into connected networks that closely paralleled and occasionally incorporated into the developing vasculature (Figure 3 and Figure 4). Retinal astrocytes form the framework that guides the structural organization of blood vessels in the developing retina [48,49]. However, it has been shown that hematopoietic cells are capable of targeting retinal astrocytes to promote both vasculogenesis and retinal vascular repair [50,51]. MACs secrete paracrine factors that recruit and guide ECFCs to areas of vasculogenesis [43]. Hematopoietic cells provide pro-angiogenic factors as well as structural support that promote angiogenesis [52]. Thus, our study adds to the increasing evidence of hematopoietic cells as key regulators of retinal vascular development.

To investigate further the potential impact of hematopoietic cells on retinal vascular development, we used VESGEN to analyze the retinal vasculature of Vav1 KO mice. Vav1 is a signal transducer that mediates cytoskeletal rearrangement required for activation and mobilization of hematopoietic cells. Phosophorylation of Vav1 is essential for cell signaling and activation of receptors in the hematopoietic system [33,53,54]. We observed a reduction in retinal vascular density in Vav1 KO mice compared to controls (Figure 5), predominantly in the medium-sized vessels (generations 4 to 6). Previous studies have shown that loss of Vav1 leads to a reduction in lymphocytes [21], particularly T cells [22,55]. Interestingly, Deliyanti et al. observed that expansion of regulatory T cells alleviates pathological angiogenesis [56], supporting the role that hematopoietic cells play in the formation and maintenance of blood vessels. In addition, Vav1 KO mice showed increased inflammation as evidenced by an increase in GFAP expression in retinal astrocytes, key modulators of retinal angiogenesis in development [50]. Thus, lack of and/or a dysfunction of hematopoietic cells in the developing retina is associated with retinal astrocyte pathology which negatively impacts vascular development. Reduced retinal vascular density is a known indicator of poor retinal function and is associated with retinal pathologies [51,57,58]. Reduced vascular density in Vav1KO mice implies a reduction in retinal blood supply of oxygen and nutrients and inadequate removal of metabolic wastes, which affects retinal function. This, in part, could account for the increase in inflammation (GFAP expression) observed in Vav1 KO retinas.

Retinopathy of prematurity (ROP) is characterized by abnormal retinal neurovascular growth in preterm newborns, caused by the exposure of the of immature retina to hyperoxic-to-hypoxic conditions leading to aberrant retinal vascularization [59,60,61,62]. The murine OIR model has been used to recapitulate and investigate the pathogenesis of ROP [63,64,65]. The OIR model, like most vascular injury models, leads to infiltration of hematopoietic cells into the retina [66,67], and intravitreally administered hematopoietic cells are able to target the retinal vasculature to reverse abnormal retinal vascular development [27]. We subjected Vav1-GFP mice to OIR injury to track the response of endogenous hematopoietic cells in pathological vascular development. Hematopoietic cells maintained close association with blood vessels as observed in non-OIR retinas (Figure 6A–C). Hematopoietic cells incorporated into blood vessels and occasionally formed ‘channels’ (Figure 6D) as framework for the formation of new vessels. These findings support that hematopoietic cells may play a vital role in orchestrating retinal vasculogenesis during development.

Our study has limitations. Even though Vav1 is widely known to be expressed in hematopoietic cells, it can infrequently be turned on in endothelial cells [68]. Nonetheless, given the overwhelming evidence in the literature that Vav1 cells are hematopoietic [55,69,70] and the retinal phenotype of the Vav1KO mice (Figure S2B), our data supports the role that hematopoietic cells play in retinal development. Data from 7-month-old Vav1 KO mice and controls (Figure S2D,E) shows that at 7 months of age, Vav1 KO retinas have a significantly reduced vascular density (Figure S2D) but no significant difference in GFAP expression (Figure S2E). Thus, while the Vav1 KO retinas recover from the increase in inflammation experienced early after birth, the loss in vascular density persists beyond the resolution of inflammation. This suggests that the reduction in vascular density is more likely due to the loss of hematopoietic cells in the retina and not secondary to inflammation. In summary, our data supports that hematopoietic cells play a vital role in orchestrating retinal vasculogenesis during development. Further studies should also seek to correlate the reduced vascular density observed in these mice with results from physiological tests of functions such as electroretinography.

## 5. Conclusions

Our study characterizes hematopoietic cells recruited into the developing retina and the possible roles of these cells in the formation of a healthy retina. In addition to microglia which are seeded prenatally, monocytes and MACs participate in the growth and development of the retina. Hematopoietic cells maintain a close association with the developing vasculature, contributing to the regulation of angiogenesis either directly or indirectly by targeting retinal astrocytes. Further investigation is required to elucidate the interplay between hematopoietic cells, astrocytes and endothelial colony forming cells in vasculogenesis.

## Data Availability

The original data presented in the study are available on request from the corresponding author.

## Supplementary Materials

The following supporting information can be downloaded at https://www.mdpi.com/article/10.3390/cells11203207/s1, Figure S1: Distribution of hematopoietic cells in retina development, Figure S2: Hematopoietic cells play a role in vasculogenesis in retina development.
