# Supplementary material for: Hematopoietic Cells Influence Vascular Development in the Retina
Source: Cells. 2022 Oct 13;11(20):3207. doi: 10.3390/cells11203207 (PMC9601270; doi:10.3390/cells11203207)
Supplement: Supplementary file 1 [file cells-11-03207-s001.zip › cells-1930189-supplementary.pptx]

## Slide 1
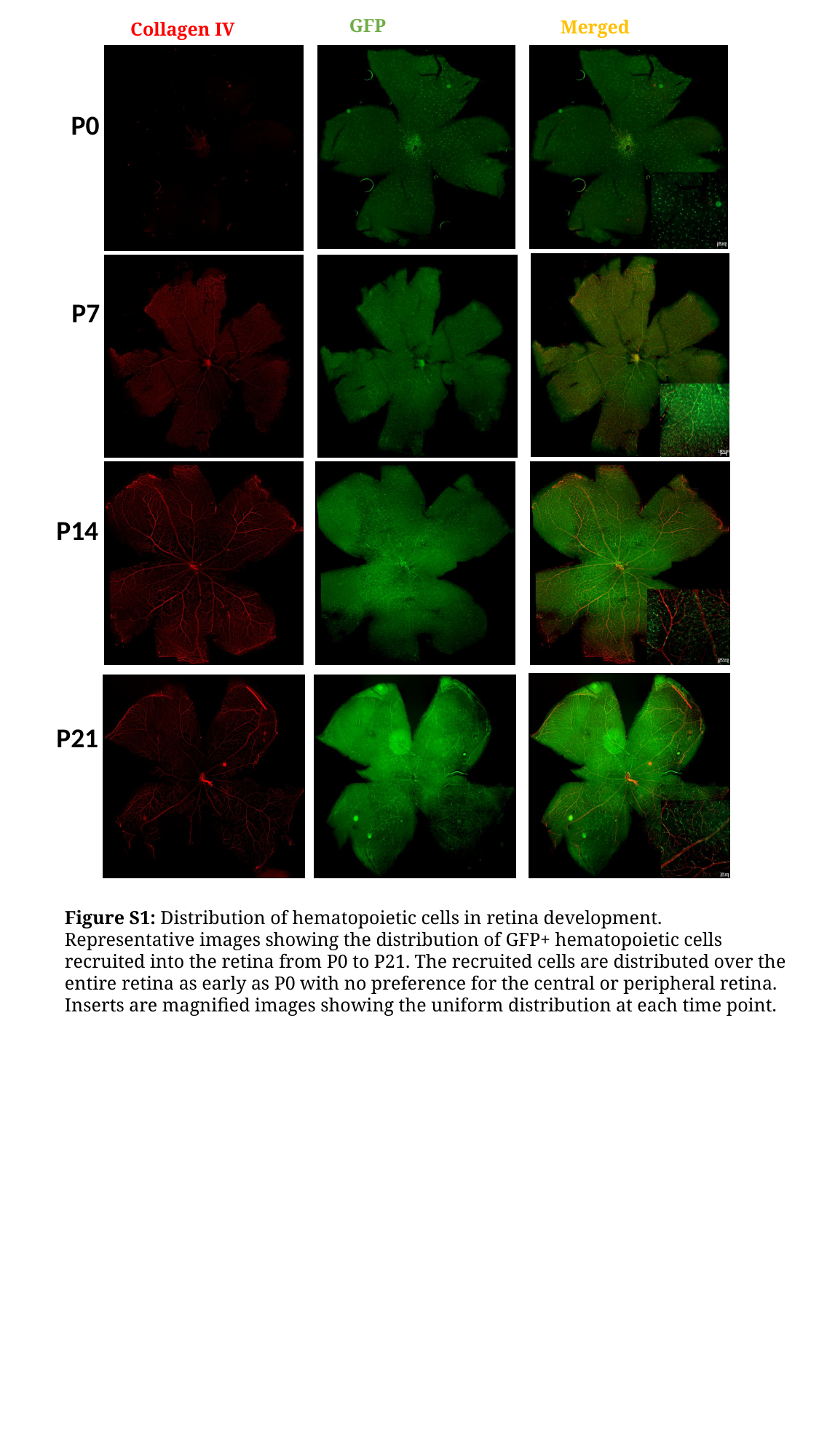

GFP
Merged
Collagen IV
P0
P7
P14
P21
Figure S1: Distribution of hematopoietic cells in retina development. Representative images showing the distribution of GFP+ hematopoietic cells recruited into the retina from P0 to P21. The recruited cells are distributed over the entire retina as early as P0 with no preference for the central or peripheral retina. Inserts are magnified images showing the uniform distribution at each time point.

## Slide 2
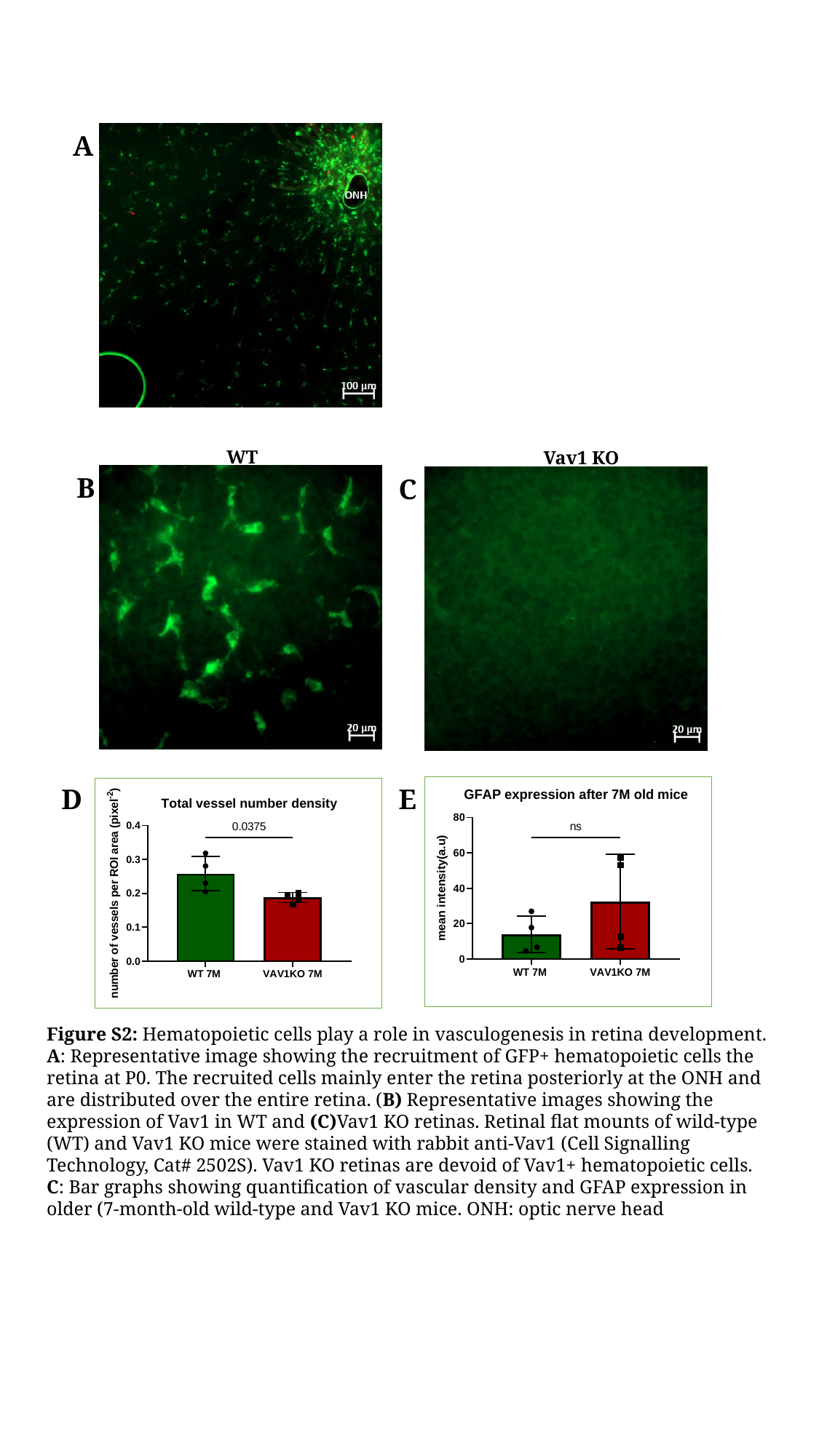

A
WT
Vav1 KO
B
C
D
E
Figure S2: Hematopoietic cells play a role in vasculogenesis in retina development. A: Representative image showing the recruitment of GFP+ hematopoietic cells the retina at P0. The recruited cells mainly enter the retina posteriorly at the ONH and are distributed over the entire retina. (B) Representative images showing the expression of Vav1 in WT and (C)Vav1 KO retinas. Retinal flat mounts of wild-type (WT) and Vav1 KO mice were stained with rabbit anti-Vav1 (Cell Signalling Technology, Cat# 2502S). Vav1 KO retinas are devoid of Vav1+ hematopoietic cells. C: Bar graphs showing quantification of vascular density and GFAP expression in older (7-month-old wild-type and Vav1 KO mice. ONH: optic nerve head
